# Collaborative Cross and Diversity Outbred data resources in the Mouse Phenome Database

**DOI:** 10.1007/s00335-015-9595-6

**Published:** 2015-08-19

**Authors:** Molly A. Bogue, Gary A. Churchill, Elissa J. Chesler

**Affiliations:** The Jackson Laboratory, 600 Main Street, Bar Harbor, ME 04609 USA

## Abstract

The Mouse Phenome Database was originally conceived as a platform for the integration of phenotype data collected on a defined collection of 40 inbred mouse strains—the “phenome panel.” This model provided an impetus for community data sharing, and integration was readily achieved through the reproducible genotypes of the phenome panel strains. Advances in the development of mouse populations lead to an expanded role of the Mouse Phenome Database to encompass new strain panels and inbred strain crosses. The recent introduction of the Collaborative Cross and Diversity Outbred mice, which share an extensive pool of genetic variation from eight founder inbred strains, presents new opportunities and challenges for community data resources. A wide variety of molecular and clinical phenotypes are being collected across genotypes, tissues, ages, environmental exposures, interventions, and treatments. The Mouse Phenome Database provides a framework for retrieval, integration, analysis, and display of these data, enabling them to be evaluated in the context of existing data from standard inbred strains. Primary data in the Mouse Phenome Database are supported by extensive metadata on protocols and procedures. These are centrally curated to ensure accuracy and reproducibility and to provide data in consistent formats. The Mouse Phenome Database represents an established and growing community data resource for mouse phenotype data and encourages submissions from new mouse resources, enabling investigators to integrate existing data into their studies of the phenotypic consequences of genetic variation.

## Introduction

Understanding the causes of variation in complex disease-related phenotypes, how these traits relate to one another, and which phenotypic outcomes most resemble human disease requires detailed characterization and integration of data across phenotyping domains. Deep phenotyping of model organisms is a powerful approach to basic and translational research of human disease. The laboratory mouse is an especially efficient and versatile model system. Mice have a relatively short lifespan, 99 % of mouse genes are shared with humans (Boguski 2002), and a rich repertoire of phenotyping modalities is available to study the physiology, behavior, and genetics of mice in normal development, aging, and disease. Mice present many advantages over direct study of diseases in humans including precise control of experimental conditions, low costs, access to tissues and interventions, and repeatability of experimentation. As a result, the laboratory mouse remains the most widely studied and most well-characterized model organism.

Thousands of inbred and genetically modified strains are currently available as live animals or as cryopreserved stocks, and more are being created and phenotyped (Ringwald et al. 2011; Brown and Moore 2012). Crosses of inbred mouse strains have revealed the genetic basis of numerous complex traits through quantitative trait locus (QTL) mapping (for numerous examples, see QTL Archive at phenome.jax.org). New genetic reference populations have been developed including the Collaborative Cross (CC) inbred strains (Churchill et al. 2004; Chesler et al. 2008; Iraqi et al. 2008; Morahan et al. 2008; Welsh et al. 2012; Threadgill and Churchill 2012) and their complementary high-precision mapping population, the Diversity Outbred (DO) mice (Churchill et al. 2012; Svenson et al. 2012a; Chesler 2013). The genomes of many widely used mouse strains, including the founders of the CC and DO, have been fully sequenced (Keane et al. 2011; Yalcin et al. 2012; Wong et al. 2012; Ananda et al. 2014), and high-density genotyping arrays are available (Yang et al. 2009).

Unlike the human population, genetic variation present in the mouse has been stabilized, characterized, and segregated (both randomly and non-randomly) across a variety of different populations. The shared genetic variation in mouse populations provides a basis for data integration and a means to discover the causal genetic variants for disease-related phenotypes. Dense genotyping and sequencing technologies enable characterization of genomic similarity of individual mice and strains. Our ability to relate this complete picture of genetic variation to phenotypic observations of individual mice enables identification and validation of the genetic basis of complex, disease-related traits, increasingly with single nucleotide resolution.

Collecting data on widely used populations provides significant opportunities to extend findings through data reuse but only if the data are harmonized and integrated. Dissemination of primary mouse phenotype data is an imperative complement to research publications; however, additional effort—beyond releasing data in supplemental files—is needed to facilitate coherent integrative analyses across multiple studies.

Primary data access is crucial for three reasons: (1) integrative analysis to find consensus among diverse studies, (2) reanalysis in light of new developments, and (3) reproducibility. Unfortunately, phenotypic data often exist in diverse and sometimes non-computable stores with insufficient documentation and restricted access. With increasing potential for data integration to provide new insights, it is critical that we provide access to carefully curated data in standardized formats that allow researchers to build upon previous studies. Recent advances in meta-analysis techniques have demonstrated the value of combining primary data across multiple mouse studies (Kang et al. 2014; Bubier et al. 2014).

## The Mouse Phenome Database

The Mouse Phenome Database (MPD; http://phenome.jax.org) (Grubb et al. 2014) stores harmonized primary data, including per mouse phenotypes acquired over multiple trials, measures, or conditions. Now in its 14th year, MPD collects, annotates, and disseminates quantitative phenotype data and protocols in an integrated relational database to facilitate faceted search and other capabilities. MPD provides a repository of mouse phenotype data and a suite of tools for comparative and quantitative analysis. Originally developed as a repository for phenotype data collected on a small and defined set of inbred strains (Paigen and Eppig 2000), the scope and role of MPD as a general repository for primary data collected on individual mice has expanded. MPD data are organized around a catalog of phenotype ontology terms; assays and protocols are extensively documented; analysis tools provide summary statistics and data visualization; and, importantly, the common data framework enables easy data access to and integration of data from multiple labs and experiments. Data come from investigators around the world and represent a broad scope of physiological and behavioral traits in naïve mice and those exposed to drugs, environmental agents, or other treatments. Access to phenotype data and protocols from different sources enables researchers to reproduce experiments; reanalyzes data with new algorithms and up-to-date bioinformatics resources; and discovers unexpected relationships among trait data that were collected in different times and places. The high standards of documentation and curation and the stability of the program at The Jackson Laboratory have made MPD a primary resource for investigators to archive and retrieve quantitative mouse phenotypic data and protocols.

## Collaborative Cross strains, Diversity Outbred mice, and related populations

**Fig. 1:**
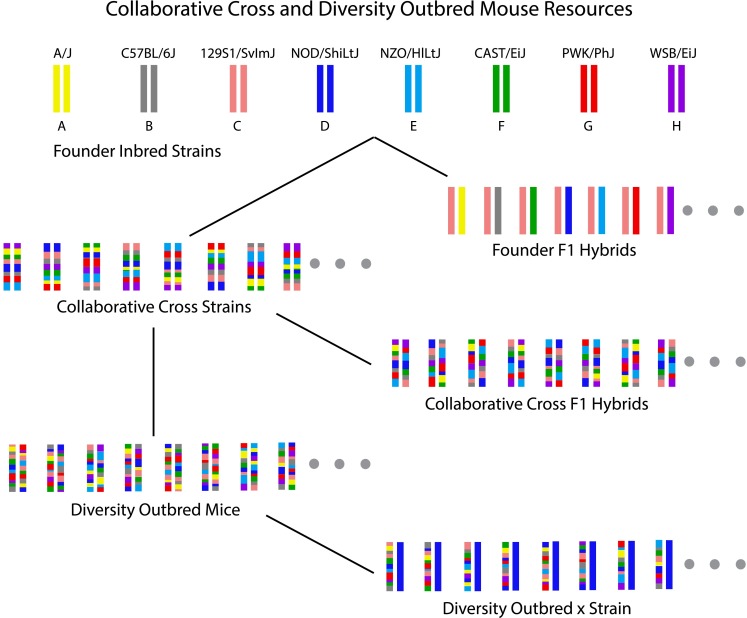
Collaborative Cross (CC) and Diversity Outbred (DO) mouse resources. The eight founder strains are depicted at the *top* as color-coded chromosomes. Color-coding for these strains has been agreed upon by the research community and standardized. Founder F1 hybrid offspring are produced from crossing two founder strains and are thus reproducible. Theoretically, there are 102 possible F1 hybrids (including reciprocal crosses). CC strains are inbred (homozygous at most loci) and reproducible. CC F1 hybrids are offspring produced from crossing two CC strains and are thus reproducible. There are many possibilities for CC F1 hybrids. DO mice were derived from CC strains through strict breeding protocols to maintain heterogeneous stocks and are thus not reproducible. Theoretically, there is an unlimited supply of DO mice. They are typically tested as a population of over 100 unique mice. “DO × strain” mice are produced from a DO mouse crossed to an inbred strain. Because DO mice are not reproducible, “DO × strain” mice are likewise not reproducible

Genetic reference populations have defined genomes, can be retrieved or reproduced indefinitely, and enable data integration through genetic correlation across genome-matched individuals or strains. The Collaborative Cross, a multi-parent recombinant inbred strain panel was created to provide greater allelic diversity and better randomization of genotypes and genetic recombinations than was previously available in genetic reference populations and thereby to improve the power, precision, and accuracy of genetic mapping and phenotype correlation analysis (Threadgill et al. 2002; Churchill et al. 2004; Chesler et al. 2008; Iraqi et al. 2008; Morahan et al. 2008). The eight founder strains of the CC include three wild-derived strains to maximize diversity (Fig. 1). This population structure reflects the design characteristics for CC strains (CCC 2012). Dense genotyping and haplotype reconstructions of the CC inbred strains are freely available (for review, see Morgan and Welsh 2015, this issue) and, together with the founder genome sequences, represent a comprehensive knowledge of these stable, reproducible genomes. There are currently about 150 extant CC strains. Each strain can provide genetically identical mice to support as many different studies as resources allow. Phenotypes collected on CC mice are directly cumulative, facilitating the comparison of new and historical data. An impressive range of phenotypic diversity has been reported recently for traits including susceptibility to melanoma (Ferguson et al. 2015), allergic airway responsiveness (Kelada et al. 2014), immune cell counts (Phillippi et al. 2014), and susceptibilities to H1N1 (Ferris et al. 2013), Ebola (Rasmussen et al. 2014), West Nile Virus (Graham et al. 2015), and *Klebsiella* (Vered et al. 2014). The inbred CC strains are limited in mapping resolution and power by the number of extant strains, and do not enable the detection of non-additive effects due to the lack of heterozygosity.

Diversity Outbred (DO) mice are derived from incipient Collaborative Cross strains and represent a complementary resource with the same allelic diversity as the CC strains, maintained in a heterogeneous stock (Churchill et al. 2012; Chesler 2013). This population is an ideal resource for genetic mapping and selective breeding studies. The high fecundity and large stock population enables sampling of extremely large numbers of unique genomes derived from the same eight founders. Over successive generations, the recombination frequency increases, resulting in ultra-high-precision mapping studies (Svenson et al. 2012a; Logan et al. 2013b; Recla et al. 2014; Kelada et al. 2014; Smallwood et al. 2014; Gatti et al. 2014; Church et al. 2014; French et al. 2015; Church et al. 2015). Each animal is genetically unique with high levels of heterozygosity, enabling precise estimation of QTL location and allelic effect. Each DO mouse requires genotyping (see below) and haplotype reconstruction for QTL analysis (Gatti et al. 2014). CC strains and DO populations have been used to map QTLs to intervals that are less than 5 and 2 Mb, respectively (Philip et al. 2011; Logan et al. 2013b).

Integration of data from DO phenotyping studies is not as direct as with the CC strains. Aggregation and integration of data across DO studies can be achieved through common phenotypes and through shared genotypes at specific genetic loci. DO mice provide a unique opportunity to evaluate the effects of a causal genetic variant in the context of a variety of genetic backgrounds.

In addition to CC inbred strains and individual DO mice, there are numerous possibilities for study of related genotypes (Fig. 1) including the eight founder inbred strains, F1 hybrids of the founder strains, F1 hybrids of CC strains (CC-RIX), and DO mice backcrossed to inbred strains. Recombinant inbred crosses of CC-RIX provide genetically retrievable and defined F1 hybrids of CC strains, which carry all the benefits of inbred genetic reference populations with the added benefits of heterozygosity. The mapping resolution of CC-RIX is theoretically identical to that of a population of CC strains due to the fixed location of recombinations in the population. Together, these advanced mouse populations make a rich and powerful genetic resource with the potential to refine trait correlations and improve trait mapping. Founder strains and DO mice are available through The Jackson Laboratory (http://www.jax.org/); CC strains are available through the UNC Systems Genetics website (http://csbio.unc.edu/CCstatus/).

## Data resources for the CC and DO

Phenotype data from early CC and DO studies have been disseminated through a variety of platforms including the MPD. In order to reap the benefits of integrated access, MPD will collect and harmonize these data in a common repository. There, the new data can be directly integrated with the QTL Archive (phenome.jax.org) and inbred strain phenotypes that make up the original MPD data resource. Integration of data from the CC and DO together with data from inbred strains, crosses, and other sets of genetically defined mice provides an opportunity for new discoveries of the function of the vast numbers of genetic variants available in laboratory mice. Access to primary data from these populations will enable and encourage reanalysis of historical data.

Mouse Phenome Database (MPD) enables users to share and reanalyze data from the CC and DO and to compare results with those obtained on other mouse strains and strain panels. MPD currently houses primary phenotype data from CC founder strains, F1 hybrids of founder strains, incipient CC strains, and DO mice covering a range of phenotypic domains, including behavior, hematology, renal function, disease susceptibility, and exercise and endurance. Additional datasets will be added to MPD as they become available. Users of these resources are encouraged to contact us about potential data submissions (see below).

**Fig. 2:**
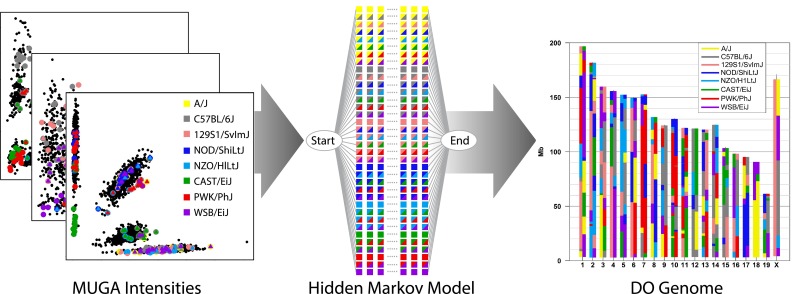
DO genotyping. High-density genotyping platform—mouse universal genotyping arrays (MUGA)—have been developed to assay the genetic diversity of CC and DO mice. The raw intensity data (*left*) from genotyping arrays provide the additional information that is not available from the binary SNP genotyping calls. Specialized Hidden Markov Model software (*center*) has been developed to recover the parental haplotype block structure of individual mice (*right*). This example is for a DO mouse

In addition to phenotype data, MPD maintains genotype data for the 8 CC founder strains, including 68 + million genomic locations (Yalcin et al. 2012; Keane et al. 2011; Wong et al. 2012) and 470,000 + locations for the mouse diversity array (Yang et al. 2009). We are expanding the database to accommodate new CC and DO genotype data collected on the “MUGA” Mouse Universal Genotyping Arrays. DO genotypes are derived from primary array intensity data using a Hidden Markov Model to reconstruct a founder haplotype mosaic (Fig. 2). Genotype data are stored as genotype calls at the probe level and as reconstructed genotype probabilities. The latter, together with the founder genomes, can be used to impute the genomes of individual DO mice with high accuracy. In addition to MPD resources, primary and derived genotype data and information on the CC inbred strains can be obtained from a number of resources described in Morgan and Welsh, 2015, including the UNC resource at http://csbio.unc.edu/CCstatus/, and founder genome sequences can be accessed at http://www.sanger.ac.uk/resources/mouse/genomes/.

## MPD visualization and analysis tools

From the MPD homepage, users can search or navigate data by strain, strain panel, investigator, phenotype, or ontology terms. There are several options for downloading data—from a single measurement to bulk download of the entire database. Video tutorials and tool demos are available to walk users through the most common tasks.

### CC founder F1 hybrids

**Fig. 3:**
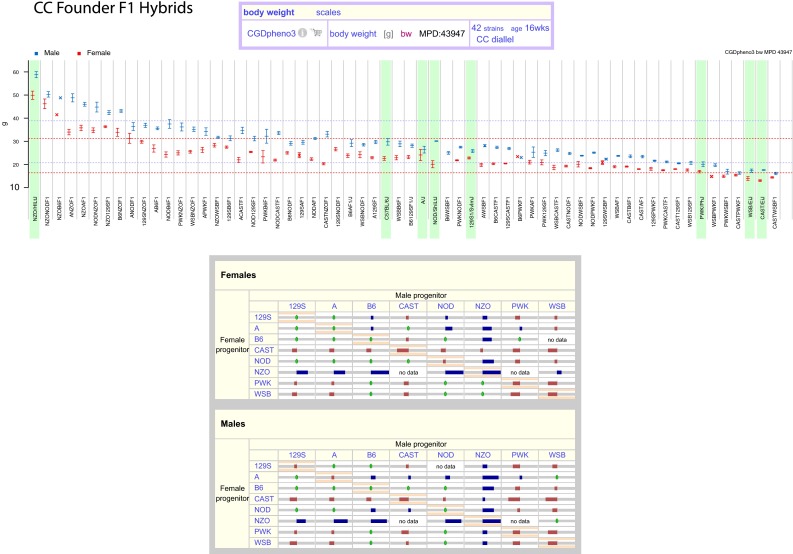
CC Founder F1 Hybrids. Data for the eight CC founder strains (*green highlight*) and F1 hybrids can be viewed in multiple ways. The *upper panel* shows a measurement plot with strain on the x-axis and body weight on the y-axis. Males are in blue and females in red (mean ± SEM). The overall mean (by sex) and ± one standard deviation are indicated by *dotted lines*. There are plotting options available for this tool, including output format (here we used eps). The *lower panel* illustrates a z-score matrix of the outcome of pairwise matings for body weight. Female offspring are shown in the top matrix and male offspring in the lower matrix. Within each matrix, the female progenitor strain is shown in the first column and the male progenitor strain is shown in the first row. Matings between individual inbred strains (on the diagonal) were conducted at the same time F1 hybrids were generated. In this case, *blue* indicates high-end outliers (*Z* > 1.0), *red* indicates low-end outliers (*Z* < −1.0), and *green* indicates average (within ± 1 standard deviation). “No data” indicate instances where offspring could not be generated. This body weight data is from MPD project CGDpheno3 (Svenson et al. 2012b)

Summary data from CC founder F1 hybrids can be viewed graphically as illustrated in Fig. 3 (upper panel). The eight founder strains, highlighted in green, exhibit a wide range of genetic diversity for body weight with strain means ranging from 13 g (CAST/EiJ females) to 58.8 g (NZO/HlLtJ males). We see that the body weights of F1 hybrids are intermediate between the extremes of founder strains. The same phenotype data can be viewed as a diallel structure using a visual “z-score matrix” to show the relative body weights of offspring from each pairwise cross, (Fig. 3 lower panel). This unique visualization reveals trends in the data; for example, F1 hybrids from male or female NZO/HlLtJ tend to weigh considerably more than other F1 hybrids while matings involving wild-derived inbred strains such as CAST/EiJ, PWK/PhJ, and WSB/EiJ tend to produce offspring with lower body weights.

### CC strains

**Fig. 4:**
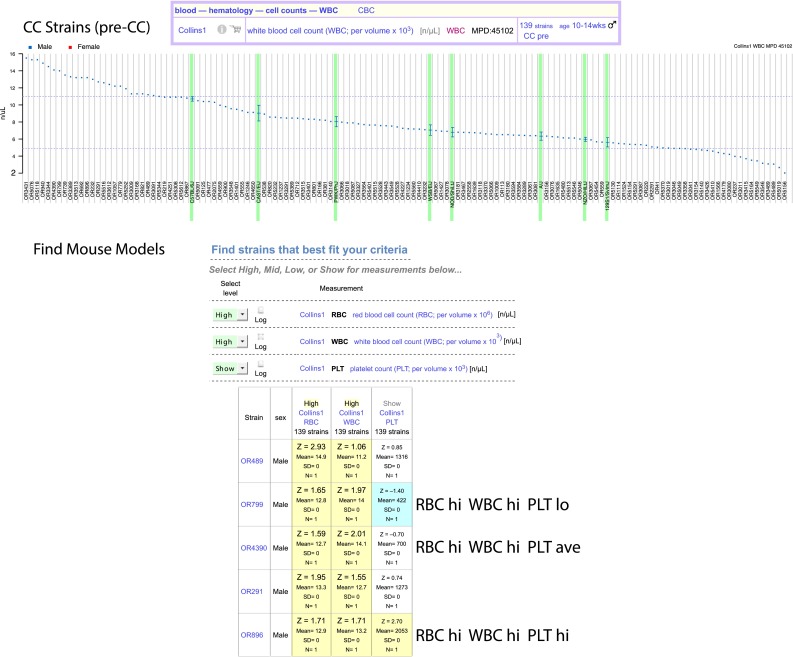
CC Strains. Data for CC strains can be viewed in a measurement plot as shown in the *upper panel* for white blood cell count (WBC), where strains are along the x-axis and WBC is on the y-axis. In this example, only males were tested (*blue*). There are options for plotting these results as well as output options (here we chose eps). The *lower panel* illustrates the criteria-fit tool. In this example, we wanted to identify CC strains with high red blood cell count (RBC) and high WBC, but with a wide range of values for platelet count (PLT). We first selected our measurements of interest (this step not shown) and applied the tool. Then we selected “High,” “Mid,” “Low,” or “Show” for each measurement according to our criteria. Results (truncated) are shown in a screenshot where high-end outliers are in *yellow* and low-end outliers are in *blue*. These blood cell parameters are from MPD project Collins1 (Collins 2012; Kelada et al. 2012)

CC data can be summarized visually as shown in the upper panel of Fig. 4. In this example, variation in white blood cell count (WBC) across 139 incipient CC strains from different lineages exceeds the range of values observed in the founder inbred strains (highlighted in green). To identify strains of interest, users can employ a criteria-fit tool to quickly find strains exhibiting specific phenotype profiles, (lower panel of Fig. 4). Here, we wanted to find strains with high red blood cell count (RBC) and high WBC, but with one strain having elevated amounts of platelet counts (PLT) and another strain having lower than normal PLT. This tool allows users to specify characteristics in up to 15 measurements that can come from multiple studies.

### DO mice

**Fig. 5:**
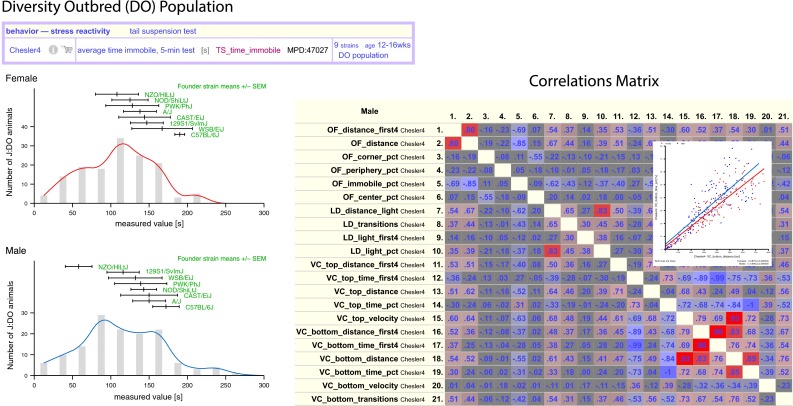
Diversity Outbred (DO). Individual measurements from DO populations can be viewed as a histogram as shown in the *left panel*. Females (*red*) are above and males (*blue*) below. The founder strains (*green*) are indicated above the histogram (mean ± SEM). There are plotting options available for this tool as well as output options (here we used eps). The *right panel* shows a screenshot of a correlations matrix for all pairwise measurements. *Red* values indicate positive correlations, *blue* indicated negative. The more intense the color, the stronger the correlation. Users can view detailed scatterplots (*inset*) by clicking on cells of the matrix. Correlation coefficients and p values are provided just below the scatterplot. Plot options are available for this tool. Data are from MPD project Chesler4 (Logan et al. 2013a, b)

Individual measurements from DO mice are summarized visually as shown in Fig. 5 (left panel) in a histogram for the tail suspension test (time immobile). Approximately 300 DO mice were tested in this study. Mean and standard error are shown for each of the eight founder inbred strains above the plot (in green). As observed with CC strains in Fig. 4, the eight founder strains exhibit less phenotypic diversity when compared to the DO population. Another view shows correlations for all pairwise measurements in a study (right panel of Fig. 5). This view helps users elucidate relationships among traits. The inset shows a scatterplot, which can be viewed by clicking on any cell of the correlation matrix.

Each of the examples above represents a means to review and interpret the results of phenotypic characterization within populations. Several compelling studies have identified genetic variants through the integration of experiments across multiple mapping populations. These include multi-population studies of HDL levels (Burgess-Herbert et al. 2008), efforts to integrate recombinant inbred and standard inbred populations in a single analysis (Bennett et al. 2010; Ghazalpour et al. 2012), and evaluations of SNP segregation patterns across overlapping QTLs (Bubier et al. 2014). Exciting developments in genetic analysis and computation have greatly increased the rigor with which investigators can combine studies across populations through the integration of population structure into mapping analysis (Devlin and Roeder 1999; Yu et al. 2006). Further, the availability of large numbers of densely genotyped strains and deep sequencing for many other strains renders feasible the reconstruction, albeit coarse in some cases, of virtually any mouse genome. With a common framework of sequence variation, it is now possible to integrate a much broader range of mouse phenomic studies, enabling discovery of the role of millions of genetic variants in the development of phenotypic variation and disease. The Collaborative Cross and Diversity Outbred population provide mouse resources with the power, precision and diversity to characterize the roles of these variants in molecular regulation, biological processes, and whole-organism disease-related phenotypes.

## Future directions

**Fig. 6:**
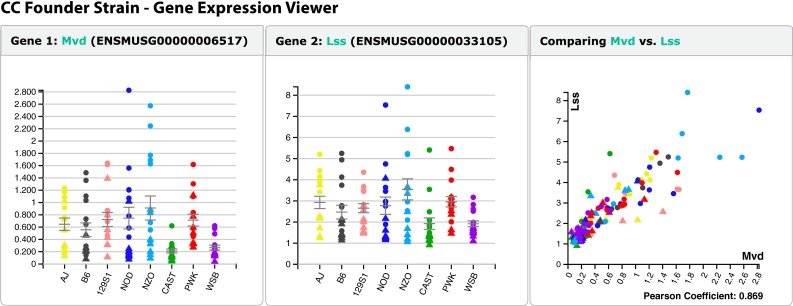
CC founder strain—gene expression viewer at do.jax.org. Users are able to input their gene of interest (in this case, *Mvd*) and generate a plot of the results across all founder strains (*left panel*) through programmatic access to the underlying data. Users can also query the database to find the top 100 correlations between their gene of interest and other gene expression profiles. In this case, the top gene found was *Lss* (*middle panel*). A scatterplot is available to view the gene expression relationship, with Pearson correlation coefficient provided (*right panel*)

MPD is being redeployed in a modern software engineering framework to facilitate the incorporation of additional data analysis modules and displays, including those specialized for access and analysis of variation in the Collaborative Cross and Diversity Outbred population. Keeping abreast of new developments in database and computer networking technology, the upcoming release of MPD will feature an applications programming interface (API) to allow programmatic access to the underlying data for analysis and display in the context of other web-based resources. In place of the current duplication of data that exists to support multiple websites, e.g., do.jax.org, access to a common source of reference data through the API will help ensure the integrity and consistency of data while giving developers the freedom to display and disseminate data in different formats and contexts. For example, see Fig. 6.

## Contributing data

To submit data from mapping and reference populations for curation, integration, and community access through MPD, please contact us at phenome@jax.org. We acquire new data generated by members of the community; incorporate evolving technologies for archiving, integrating, and analyzing new and existing data; and expand activities that promote research reproducibility within and across resources.
